# Enhancement and validation of a quantitative GC–MS method for the detection of ∆9-THC and THC—COOH in postmortem blood and urine samples

**DOI:** 10.1016/j.mex.2024.102962

**Published:** 2024-09-19

**Authors:** Somayeh Paknahad, Farzaneh Jokar, Mohammad Kazem Koohi, Masoud Ghadipasha, Jalal Hassan, Maryam Akhgari, Mehdi Forouzesh

**Affiliations:** a Division of Toxicology, Department of Comparative Bioscience, Faculty of Veterinary Medicine, University of Tehran, Tehran, Iran; b Legal Medicine Research Center, Legal Medicine Organization, Tehran, Iran

**Keywords:** Cannabinoids, ∆9-tetrahydrocannabinol, Urine, Blood, Post-mortem, GC–MS, Forensic toxicology, LLE

## Abstract

Cannabis is frequently detected in forensic investigations and is associated with an increased risk of fatal car crashes. This study aims to develop a method to detect and measure ∆9-tetrahydrocannabinol (∆9-THC) in blood and its metabolite, 11-nor-9-tetrahydrocannabinol-carboxylic acid (∆9-THC—COOH), in urine. The procedure employs two liquid-liquid extraction methods in conjunction with GC–MS in SIM mode. Both compounds were successfully processed, demonstrating the method's ease of use and efficiency. The method was validated for selectivity, identification capability, linearity, precision, limit of detection (LOD), limit of quantification (LOQ), and accuracy. Its effectiveness was further demonstrated by applying it to 30 authentic urine and blood samples from cannabis-related cases, establishing it as a valuable option for routine cannabinoid analysis in forensic toxicology labs.

• The linearity range was 25–300 ng/mL for ∆9-THC in blood, and 50–300 ng/mL for ∆9-THC—COOH in urine, and calibration curves for both analytes showed R² values consistently above 0.989, confirming their linearity.
• The LOD and LOQ for THC—COOH in hydrolyzed urine were 25 ng/mL and 50 ng/mL, respectively, and for THC in blood, they were 15 ng/mL and 25 ng/mL, respectively.
• The variation coefficients were below 14%, and recoveries exceeded 81% for both compounds.

## Graphical abstract

**Figure:**
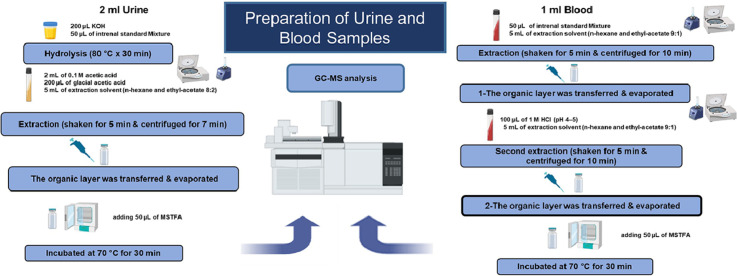

Specifications table

**Table:** 

Subject area:	Pharmacology, Toxicology and Pharmaceutical Science
More specific subject area:	Analysis
Name of your method:	LLE
Name and reference of original method:	LLE
Resource availability:	It is not applicable.

## Background

The most widely used illicit substance globally is cannabis, which contains a high concentration of ∆9-tetrahydrocannabinol (THC) [1]. Recent reports have indicated a rise in cannabis seizures and usage in Iran, particularly among the youth [2]. A study published in 2023 by (INCAS) the National Center for Addiction Studies of Iran (INCAS) found that two-thirds of cannabis users aged 15 to 64 had experienced at least one unfavorable health-related event in the previous six years [3]. The trend observed in Iran may be influenced by the acceptance of cannabis as a recreational drug in other countries [4]. Furthermore, due to the risk of developing drug dependence, cannabis use can lead to serious adverse health effects. These include acute psychosis and impaired driving, both of which have been extensively documented [5]. Among the over 100 identified cannabinoids, trans-∆−9-tetrahydrocannabinol (THC) is recognized as the most potent in terms of its psychoactive effects [6].

The choice of matrix for quantitatively analyzing cannabis metabolites will depend on the study's objectives. Options include urine, blood, plasma serum, saliva, and hair, among others. The selection is influenced by the sampling method (invasive or non-invasive) and the detectability or half-life of the analytes in the chosen matrix [7].

**Fig. 1:**
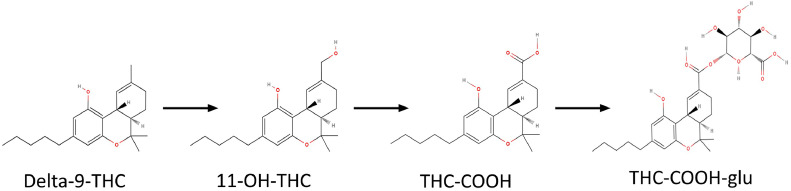
Overview of the major THC metabolites. 11-OH-THC, the product of phase I of hepatic metabolism, THC—COOH, inactive metabolite, and THC—COOH-glucuronide which is the product of glucuronidation process in phase II of hepatic metabolism excreted in urine.

In forensic toxicological drug analysis, urine and blood are commonly used biological samples due to their availability, homogeneity, and the ease with which standard procedures can be applied [8]. Delta-9-tetrahydrocannabinol (Δ9-THC) is metabolized in the liver into the psychoactive compound 11‑hydroxy-THC (OH-THC), which is subsequently oxidized to form the inactive metabolite 11-nor-9-carboxy-Δ9-tetrahydrocannabinol (THC—COOH) [6]. Approximately 80–90% of a given dose of Δ9-THC is metabolized and excreted within five days, with over 65% being eliminated through feces and around 20% through urine. The major metabolite OH-THC is predominantly found in feces, while THC—COOH glucuronide is a key metabolite detected in urine. After smoking cannabis, the concentration of THC in the blood peaks within ten minutes and is rapidly distributed to various tissues due to its fat-soluble nature [9]. The elimination half-life of Δ9-THC varies from 1 to 3 days in occasional users and can extend to 5 to 13 days in chronic users. THC—COOH is the most reliable biomarker for determining the extent of marijuana smoke exposure in humans [10] Fig. 1.

The extraction of cannabinoids from biological samples is a critical step. This procedure must be highly reproducible, efficient in recovering analytes, selective, and capable of minimizing interference from the matrix [11].

To identify the total amount of THC—COOH metabolite in a urine sample, the glucuronide must first be hydrolyzed either by alkaline treatment or enzymatically. While the majority of enzyme hydrolysis techniques take a long time, taking 8–16 h, alkaline hydrolysis is preferred for breaking down THC—COOH-glucuronide due to its efficiency, repeatability, and cost-effectiveness [12,13].

When analyzing urine samples for cannabis use identification, immunoassay tests may encounter challenges such as cross-reactivity, specificity, and sensitivity. A two-step approach is often used to address these issues: an initial screening test is performed, followed by a more specific confirmatory test if the result is positive. Techniques such as gas chromatography-mass spectrometry (GC–MS) and liquid chromatography-mass spectrometry (LC-MS) are commonly employed for confirmatory analysis.

Gas chromatography-mass spectrometry (GC–MS) is favored for confirming abused drugs in urine due to its lower consumable costs. Though derivatization is necessary to analyze polar drugs and their metabolites, GC–MS is highly effective in identifying unknown substances by operating in both selected ion mode (SIM) and full scan mode. It offers high specificity, sensitivity, and a low limit of detection, making it ideal for searching mass spectral libraries [14,15]. We use the liquid-liquid extraction (LLE) method instead of conventional solid-phase extraction (SPE) methods. LLE procedures can be optimized specifically for forensic analysis [[14], [15], [16], [17]].

There are few studies focused on post-mortem analysis distribution and detection of cannabinoids [9]. Therefore, forensic toxicological analyses are especially crucial in this field.

This paper outlines two methods for detecting and quantifying THC in whole blood and THC—COOH in urine using liquid-liquid extraction (LLE). The procedure includes derivatization with (MSTFA) N-methyl-N-(trimethylsilyl) trifluoroacetamide and subsequent analysis using gas chromatography-mass spectrometry (GC–MS). Our primary goals were to improve reproducibility and optimize the extraction efficiency of these methods. This study presents the first validated GC–MS method for the simultaneous analysis of THC in blood and THC—COOH in urine following liquid-liquid extraction. Its application to 30 actual post-mortem samples obtained by our Department of Forensic Toxicology has demonstrated its effectiveness and reliability.

## Materials and methods

### Standards and reagents

Methanolic solutions of Δ9-THC and internal standard CBN (1 mg/mL) were purchased from Lipomed Standards (Basel, SWITZERLAND), Standard solutions of Δ9-THC—COOH (1.0 mg/mL in methanol) Sigma Corporation, MSTFA (N-Methyl-N-trimethylsilyl trifluoroacetamide) was sourced from Supelco® (CAS Number: 24,589–78–4) and Potassium Hydroxide pellets (Product number: 1.05032.1000), glacial acetic acid (Product number: 1.00056.2500), Hydrochloric acid (Product number:1.00317.2500), methanol (Product number: 1.06007.2500) and were all supplied by Merck® in (Darmstadt, Germany Co), N-hexane (Lot number: 84L321, CAS: 110,543) was obtained from DUKSAN® in (KYUNGGI, South Korea), ethyl acetate (Product number: A9346) was purchased from ROMIL® and Deionized water (Product number:1.1720) was provided by Neutron in (Tehran, Iran). To obtain the final 100 µg/mL concentrations, working solutions were prepared by diluting the stock solutions with methanol. Every solution was kept at −20 °C in a freezer.

### Preparation of calibration standards

The purchased stock solutions prepared a 1 mg/mL methanolic solution containing Δ9-THC for blood and THC—COOH for urine. These three working solutions prepared 100, 1000, and 10,000 ng/mL in methanol. For each analysis, calibrators of 50, 75, 100, 150, 200, 250, and 300 ng/mL of drug-free urine and 15, 25, 50, 100, 150, 200, and 300 ng/ml of drug-free blood were prepared.

### Preparation of control standards

The prepared control solutions for urine and blood contained THC—COOH and ∆9-THC at concentrations of 100 and 1000 ng/mL, respectively. For the calibration standards, three control solutions of THC—COOH at concentrations of 85, 125, and 225 ng/mL were prepared using drug-free urine. Similarly, three control solutions of Δ9-THC at 25, 180, and 250 ng/mL concentrations were prepared using drug-free blood for each analysis.

### Sample collection

Thirty urine samples that had tested positive for the presence of the THC compound using immunoassay kits in the drug detection section were set aside for quantitative measurement of THC—CCOH. The same laboratory numbers assigned to the positive urine samples were used to analyze the Δ9-THC compound in the blood samples. The urine and blood samples taken post-mortem were kept at 5 °C.

### Liquid-liquid extraction (LLE)

#### Urine samples analysis

To each calibration standard THC—COOH, 1 mL of drug-free urine samples and 50 µL of CBN (internal standard, 100 ng/mL) were added to 16 × 100 mm screw-cap glass tubes. Potassium hydroxide (200 µL of 11.8 N) was added to the mixture, which was then placed in an incubator at 80 °C for 30 min for hydrolysis. After removal from the incubator and cooling to room temperature, 2 mL of 0.1 M acetic acid, 200 µL of glacial acetic acid, and 5 mL of an extraction solvent (a mixture of n-hexane and ethyl acetate in an 8:2 vol ratio) were added to the tubes. The mixture was vigorously shaken for 5 min, then centrifuged at RCF = 1800 × *g* for 7 min to facilitate phase separation. The organic layer was transferred to a 1.5 mL GC–MS vial and dried under nitrogen at 50 °C. For derivatization, 50 µL of MSTFA (N-Methyl-N-trimethylsilyl trifluoroacetamide) was added to the dried residues. The mixture was then placed in an incubator at 70 °C and kept there for 30 min.

#### Blood samples analysis

Fifty microliters of the internal standard solution were mixed with one milliliter of blood. In a vortex shaker, liquid-liquid extraction was performed for five minutes using five milliliters of n-hexane and ethyl acetate (9:1, v/v). The organic phase was separated after centrifugation at 2800 × *g*, and the residual aqueous sample was acidified to a pH of 4–5 with 100 µL of 1 M HCl before undergoing a second extraction. A second centrifugation was then performed to obtain an additional extract. The combined organic phases were completely dried by evaporating them under nitrogen at 50 °C. The dried sample was subsequently silylated with 50 µL of MSTFA in an oven preheated to 80 °C for 30 min to facilitate derivatization. Finally, a 0.6 µL sample was added to the GC–MS splitless inlet at 280 °C.

#### GC–MS analysis

An Agilent 5975C GC-MSD single quadrupole instrument was connected to an Agilent 7890A GC system. An Agilent HP-5MS capillary column (30 m, 0.25 mm ID, 0.25 µm film thickness, Macherey-Nagel, Oensingen, Switzerland) was used for chromatographic separation of the analytes. The carrier gas, helium, was maintained at a steady 1.2 mL/min flow rate. The injector temperature was held at 250 °C and 280 °C. A splitless injection mode was employed. The MS analysis was conducted using a 70 eV electron ionization mode. The mass spectrometric detection and GC parameters were set in SIM mode (single ion monitoring) Table 1. A solvent delay of three minutes was chosen, and an injection volume of 0.5 µL was utilized.

**Table 1:** Gas chromatography parameters for ∆9-tetrahydrocannabinol (THC) in Blood and 11-nor-∆9-tetrahydrocannabinol-9-carboxylic acid (THC—COOH) in urine specimen.

Sample	GC.MS operating conditions	Target & Ions monitored (*m/z*)
Urine	Inducted into the splitless Column pressure: 5 psi Injector temp: 250 °c Purge time on: 1.0 min Detector temp: 280 °c Initial oven temp: 120 °c Initial time: 0.5 min Temperature ramp: 30 °c.min Final oven temp: 6.0 min MS source temp: 230 °c Mass mode: selected ion monitoring (SIM)	THC—COOH 371, 488, 473
		CBN (IS) 367, 368, 382
Blood	Inducted into the splitless Injector temp: 280 °c Helium flow: 1.3 ml/min Ramp1: 70 °c for 2 min Ramp2: rate 20 °C/min, 250 °C for 4 min Ramp3: rate 20 °C/min, 300 °C for 17 min Mass mode: selected ion monitoring (SIM)	Δ9-THC 371, 386**,** 303
		CBN (IS) 367, 368, 382

### Method validation

The GC–MS technique was validated in accordance with current standards [18,19]. This methodology supports pharmacokinetic studies and concentration monitoring of known analytes (i.e., dose) and their metabolites. Interferences were assessed in accordance with SWGTOX criteria.

### Specificity and selectivity

The approach's selectivity demonstrates its ability to identify the studied compounds without interference from the analyzed biological samples [20]. If the selected ions for monitoring are unsuitable, drugs and endogenous compounds may produce similar ions with the same retention times for high molecular weight compounds. To evaluate selectivity, we examined interference signals in target ions of interest at appropriate retention times for each analyte and internal standards in at least ten drug-free urine and blood samples. In addition, triplicate samples of the highest calibrator with internal standards, as well as triplicate blank samples with external standards, were tested. The study also assessed potential interference from common drugs by analyzing samples containing cocaine, cocaethylene, norcocaine, amphetamine, methamphetamine, MDA, MDMA, MDEA, tramadol, methadone, morphine, codeine, heroin, barbiturates, buprenorphine, ketamine, and 6-monoacetylmorphine, all at 1000 ng/mL. The response of any interfering peaks near the internal standard's retention time should be below 5% of the internal standard's response [15].

### Linearity and calibration model

Linearity was established by analyzing fortified specimens in the 50–300 ng/mL range for urine and 15–300 ng/mL for blood over three separate days. Seven matrix-matched calibrators were evenly spaced over the concentration range. Results were evaluated using least squares regression. Calibrators must be within 15% of the target value or 20% at the lower limit of quantification (LLOQ) and r² ≥ 0.99.

### Limits of detection and quantification

The limit of detection (LOD) refers to the lowest concentration that could be detected with a signal-to-noise ratio (SN) > 3 for all the substances being analyzed. The lowest concentration that can be precisely and reliably determined, within 20% of the target concentration and with a maximum coefficient of variation (CV) of 20%, is known as the lower limit of quantification (LLOQ). The upper limit of quantification (ULOQ) is defined as the highest calibrator.

### Intra-Day, inter-day, and intermediate precision and accuracy

Accuracy and precision within and between days were assessed using the three control samples discussed in Section 2.3. The percentage bias from the expected target concentration is used to express inter-day and intra-day accuracy, which must be within 15% of the target concentration or 20% at the LLOQ. The assay's repeatability is gauged by intra-day precision, expressed as a percentage of the coefficient of variation (CV). Inter-day precision, also known as the CV (%), gauges how repeatable the assay is between days [17,21].

### Extraction efficiency

The detector response is measured When an analyte is in excess and extracted from a biological matrix. Recovery is then determined by comparing this response to the response obtained from the actual concentration of the pure, authentic standard [22]. Analyte recovery optimization is essential to ensure effective and consistent extraction. The analyte and internal standards must have consistent and repeatable recovery rates, even though 100% recovery is not required. The analytical results of extracted samples at two different concentration levels (50 and 300 ng/mL in urine and blood samples) were compared with samples spiked with the standards following the extraction step to assess the recovery efficiency of the method. Standards that aren't extracted indicate a full recovery. To ensure it was dependable and consistent, this comparison (*n* = 3) was carried out in three days.

### Stability

The stability of cannabinoid compounds at room temperature was evaluated by testing low and high controls stored at room temperature. At each time point, triplicates of each control were removed from the freezer and kept at room temperature for 2, 6, and 24 h before extraction and analysis. Additionally, triplicate low and high controls were subjected to three freeze-thaw cycles before extraction and analysis. The stability of the treated samples was assessed by retesting the extracts derived from the low and high controls (*n* = 3) 21 days after the initial test, with the extracts stored at room temperature until the retest.

### Carryover

Analyte concentrations in negative blood or urine samples were measured immediately following each of the three injections of high-concentration (300 ng/mL) analyte blood or urine samples to evaluate instrument carryover. The results obtained from the injections of blank samples were compared with those of the limit of quantification (LOQ). The signal must not exceed the LOQ concentration.

## Results and discussion

Detecting cannabinoids is important when investigating cases where cannabis may have contributed to a person's death, such as in workplace or traffic accidents. It's difficult to determine the impact of cannabis use because establishing a link between postmortem blood and urine concentrations and the individual's condition before death is challenging. It's crucial to take into account the detection time of drugs (the duration after drug intake when they can still be detected) when examining drug levels in bodily fluids [17]. The study used a simple LLE method with the adaptation of existing techniques [14,15,23,24].

Drug detection time, also known as the duration in which a drug remains detectable after administration, is a critical factor when examining drug presence in bodily fluids. The concentrations of ∆9-THC in blood, urine, or other bodily fluids are impacted by pharmacological factors such as drug dosage, plant source, preparation method, route of administration, and metabolism and excretion rates, as well as analytical factors like the sensitivity, specificity, and accuracy of the testing method. The devised technique made it possible to determine the analytes with favorable outcomes accurately [17]. Derivatization was necessary to enhance sensitivity for ∆9-THC and THC—COOH, which exhibited poor chromatographic behavior and insufficient sensitivity if injected without derivatization, as other authors also reported [9,17,21,23].

**Fig. 2:**
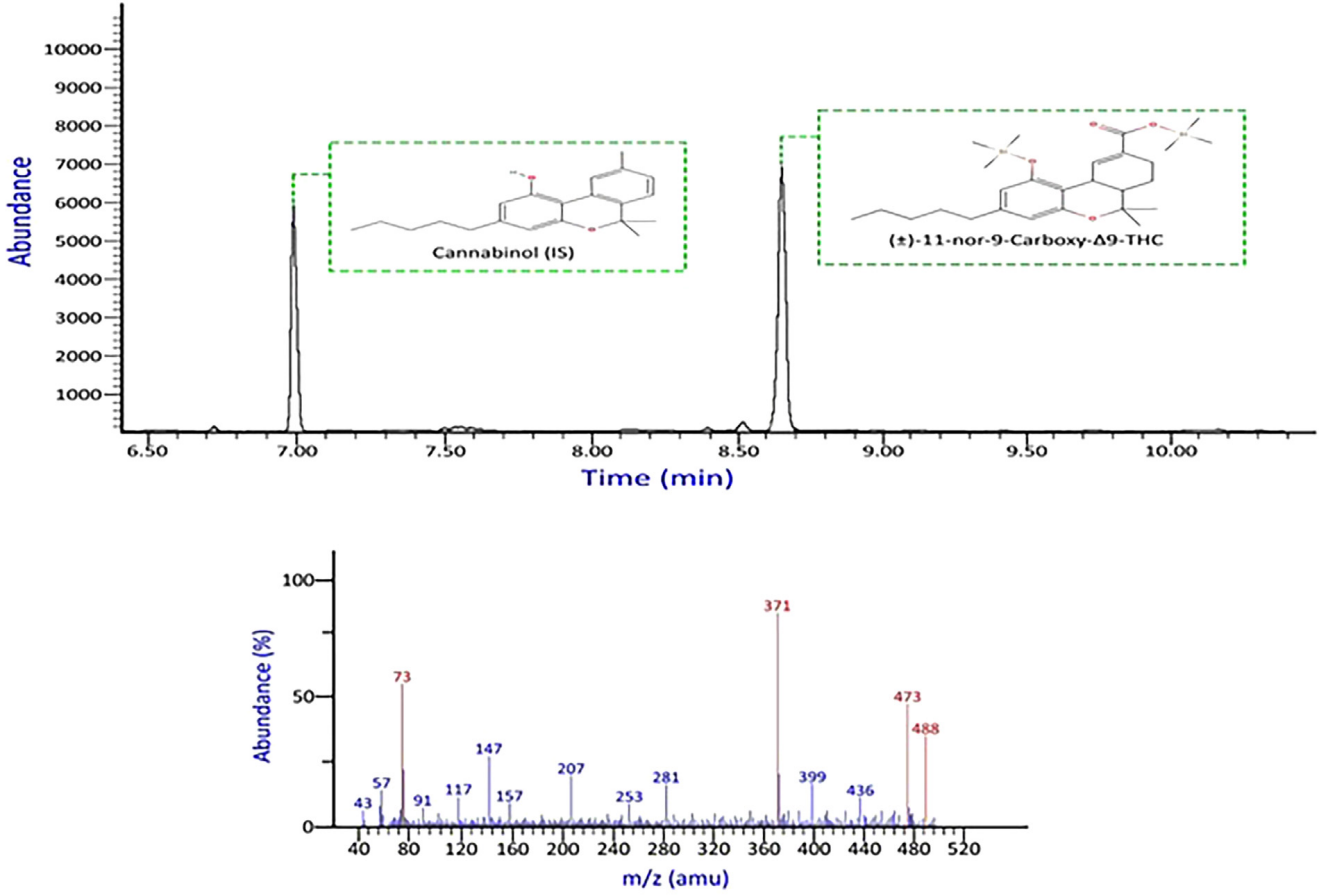
Chromatogram and mass spectral data of THC—COOH in urine sample, (selected ions *m/z* = 73, 371,473, 488), obtained using LLE/GC–MS.

**Fig. 3:**
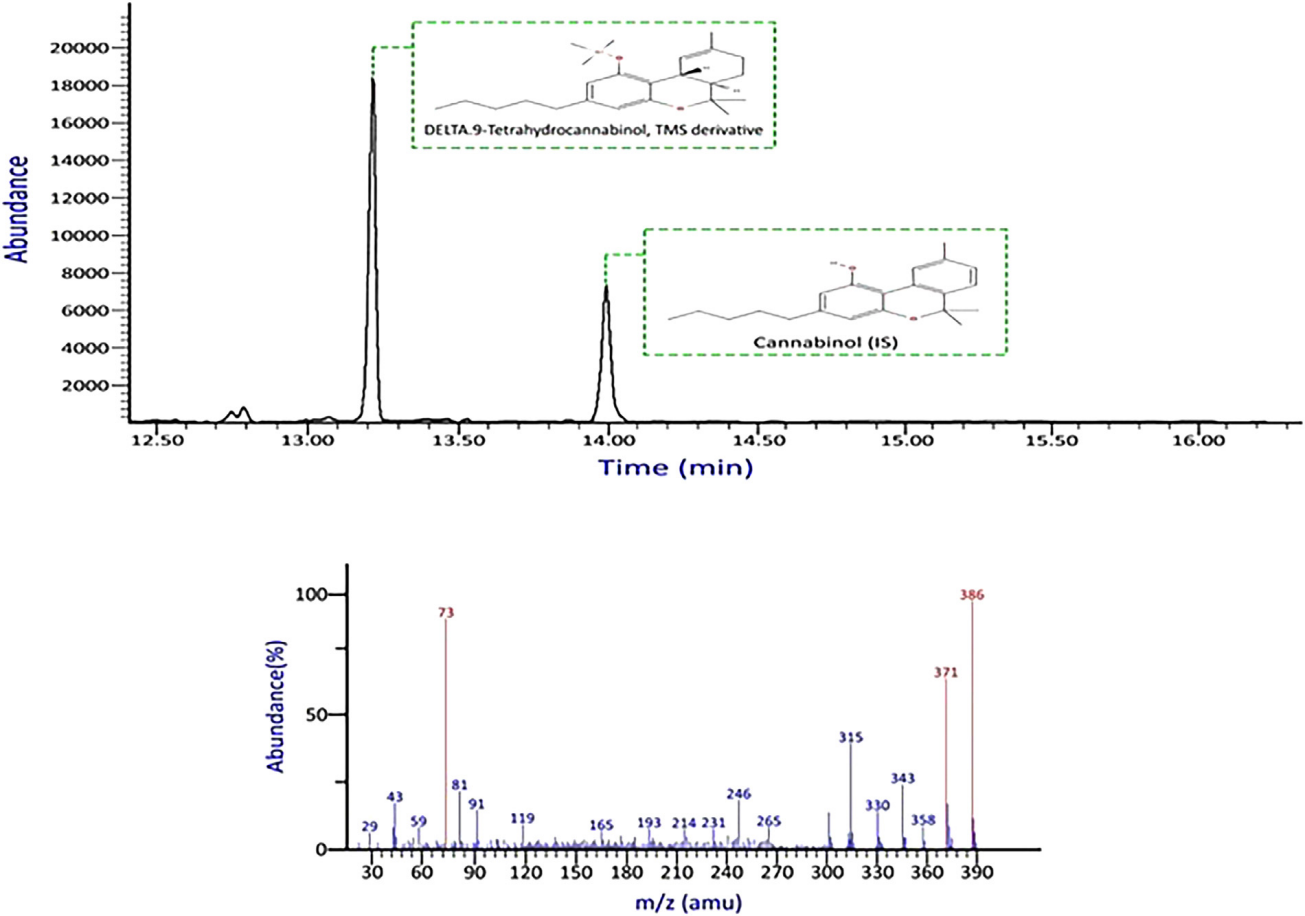
Chromatogram and mass spectral data of Δ9-THC in blood sample, (selected ions *m/z* = 73, 371, 386), obtained using LLE/GC–MS.

None of the commonly encountered compounds produced interfering signals on the ions monitored for target analytes or internal standards. There was no discernible interference from the drug-free blood and urine sources. Figs. 2, 3 show chromatograms and mass spectral data of THC—COOH and ∆9-THC.

Data were analyzed using a linear least squares (LLS) regression model. The six calibrators underwent duplicate analyses on three different days. Every calibration curve showed strong linearity (R² > 0.998) for ∆9-THC and THC—COOH. This linearity interval represents the concentration of cannabinoids typically found in users' urine and blood samples, ranging from 5 to 300 ng/mL [25]. Moreover, the LOD and LOQ were measured in both blood and urine. In hydrolyzed urine, the LOD and LOQ were 20 and 50 ng/mL for THC‐COOH, respectively; in blood, the LOD and LOQ were 15 and 25 ng/mL for THC, respectively Table 2. These validation results are consistent with findings from other studies that used blood and urine as biological samples [13].

**Table 2:** Limit of quantitation, lower detection limit, and calibration values for Δ9-THC—COOH in urine and Δ9-THC in whole blood.

Analyte	Linearity range (ng/mL)	Slope	Intercept	R^2^	LOD (ng/ mL)	LOQ (ng/mL)
**Hydrolyzed urine**						
THC‐COOH	50–300	0.0165	+0.0236	0.999	20	50
**Whole blood**						
∆^9^-THC	25–300	0.0063	−0.0089	0.9995	15	25

As well, the precision (CV%) and accuracy (Bias) parameters yielded satisfactory results. The CV and RSD values were less than 15% at other concentrations and less than 20% at the LOQ concentration. The precision and bias for each analyte in blood and urine were within allowable bounds for both inter and intra-day measurements Table 3. Parameters were also consistent with or better than those reported in other studies [14,17,20,26].

**Table 3:** Precision and accuracy for ∆9-THC and THC—COOH in blood and urine samples.

Analyte	Nominal concentration (ng/mL)	Intra‐day (*n* = 3)			Inter-day (*n* = 3)		
		Determined Concentration (ng/mL)	Precision (CV%)	Accuracy (%)	Determined concentration (ng/mL)	Precision (CV%)	Accuracy (%)
Hydrolyzed urine							
**THC‐COOH**	85	83.83	2.21	1.37-	83.67	2.02	−1.56
	170	171.03	1.68	0.60	170.22	1.59	0.13
	280	277.61	0.57	−0.85	276.97	0.70	−1.07
Whole blood							
**∆^9^-THC**	80	85.00	4.33	6.25	81.07	8.43	1.34
	180	178.12	0.16	−1.04	179.85	1.51	−0.07
	250	248.26	6.48	−0.69	248.95	4.20	−0.41

Papoutsis et al. reported the following results for Δ9-THC in plasma samples: R² value ≥ 0.992, intra-assay precision between 4.45% and 5.97%, and inter-assay precision ranging from 3.28% to 4.74% [27]. Karas et al. obtained the following results for THC—COOH in urine samples: R² value ≥ 0.998, with bias and precision for controls within ±14.1% and ≤9.1%, respectively, (coefficients of variation) CV≤ 9.9% [28]. However, solid phase extraction (SPE) methods used in other techniques may have increased the cost of analyses. The stability of the specimen under laboratory storage conditions is crucial for maintaining dilution integrity. With the concern that THC binds to polypropylene tubes, storage and extraction must take place in glass pipes [13,29]. For urine and blood controls, the stability of the cannabinoid compounds was assessed at 2, 6, and 24 h at room temperature Table 4. No decrease in THC in blood or THC—COOH in urine was observed in the control samples kept at room temperature prior to analysis. Additionally, three freeze/thaw cycles on control samples revealed no loss of cannabinoid compounds. The long-term stability test evaluated the time over which the matrix remains stable when stored at −5 °C. Samples were analyzed at low (100 ng/ml) and high (250 ng/ml) concentrations. After 21 days of storage, the analytes remained stable.

**Table 4:** Stability estimation results for THC—COOH and Δ9-THC in spiked postmortem urine and blood samples.

Storage conditions, analyte concentration (ng/mL)	Urine (THC—COOH)	Blood (Δ9-THC)
	(%CV)	
Room temperature, 24 h (*N* = 3)		
100 ng/mL	6.54	16.77
250 ng/mL	2.74	8.25
Freeze-thaw cycle After 3 Cycles (*N* = 3)		
100 ng/mL	7.99	11.27
250 ng/mL	2.95	6.22
Long-term, 21 days (*N* = 3)		
100 ng/mL	7.25	15.15
250 ng/mL	2.44	3.65

Drug stability, especially cannabinoids, has long been a subject of interest [1,23,29]. It's important to note that when an acidic solution is required to elute analytes, the stability of the analytes decreases as the pH value decreases [30].

When all analyte concentrations are within ±20% of the concentrations at zero time, the sample is considered stable [28], it remains stable for re-extraction purposes for at least 3 weeks under current laboratory storage conditions. These results, validated using blood and urine as biological matrices, are consistent with findings from other studies. Ferry et al. tested the sustainability of sample analytes in urine and blood prototypes within 3 days and 40 h. No significant loss of signal was observed for any analytes [1]. Höfert et al. have demonstrated stability for processed samples over 3 days, through three freeze-thaw cycles, for 1 week at room temperature, and for 1 month at 4 °C and −20 °C, with no significant interferences observed [31]. In the present study, no carryover was observed after the injection of the highest calibrator.

Consistent and efficient recovery of analytes is crucial for an excellent analytical method. Good recovery allows the technique to detect low analyte concentrations. An evaluation of the recovery showed that analyte recovery ranged from 84.97% to 90.80% for urine and 79.74% to 90.58% for blood Table 5. This successful recovery indicates that the extraction and derivatization processes did not result in a significant loss of cannabinoid analyte compounds. The recovery value in previously published studies for ∆9-THC and THC—COOH in blood and urine samples by LLE extraction ranged from 80.54% to 82.54% and 63.4% to 85.8%, respectively [17,20]. Few studies have simultaneously investigated the presence of cannabinoids in both postmortem blood and urine samples, and the way they work has varied [8,26]. Stefanelli et al. directly used the derivatizing agent propyl chloroformate (PCF) in the deproteinized blood sample and the hydrolyzed urine sample [26]. Previous reviews have often focused on a single sample type or have been performed on living individuals [13,32,33].

**Table 5:** Recovery data for ∆9-THC and THC—COOH from blood and urine samples.

Concentration (ng/mL)		Intra-day (*n* = 3)		Inter-day (*n* = 3)	
		Mean Recovery (%)	CV (%)	Mean Recovery (%)	CV (%)
THC—COOH (Urine)	85	88.19	1.98	87.88	1.78
	170	87.92	0.82	87.44	1.05
	300	89.81	0.74	88.67	2.60
∆9-THC (Blood)	80	81.08	6.51	82.09	10.77
	180	86.19	1.38	82.99	4.47
	250	86.54	4.43	89.56	3.51

**Fig. 4:**
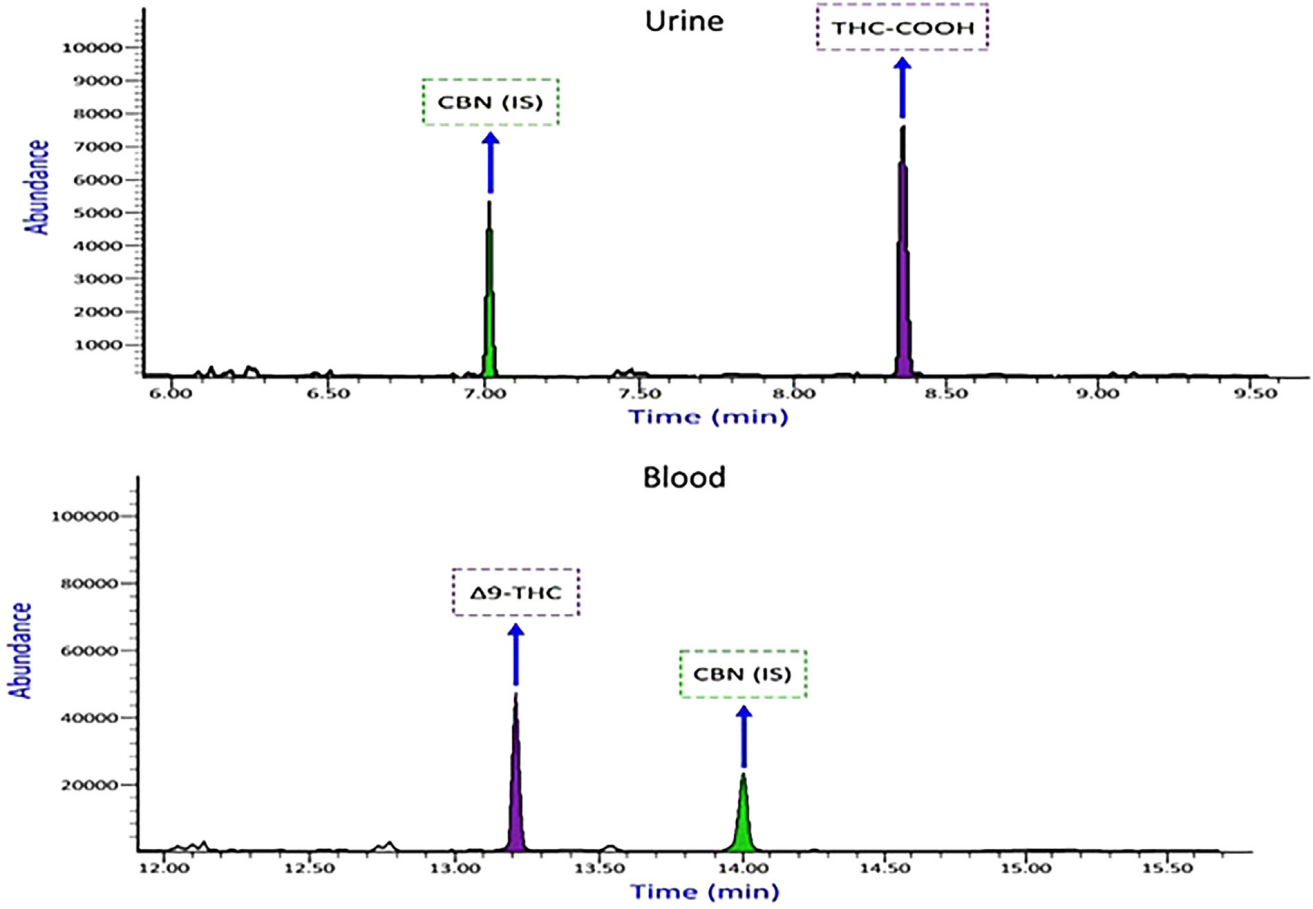
Chromatogram and mass spectral data of urine and blood samples containing 126.98 ng/mL of THC—COOH in urine and 55.84 ng/mL Δ9-THC in blood (case number 8).

This new technique could have advantages, such as reducing preparation time, the amount of MSTFA(derivatization), and the sample volume required compared to previous studies [13,15,26,27]. Fig. 4 shows the chromatogram of THC—COOH and Δ9-THC obtained from a real urine and blood sample analysis.

Thirty authentic samples of deceased individuals were submitted to our laboratory for toxicological analysis using this method Table 6. The samples were chosen after our laboratory's immunoassay screening procedure showed a positive result for Δ9-THC (cut off 50 ng/mL). Numerous scientific studies have linked road accidents as a leading cause of death among marijuana users [9,34,35]. However, our research indicates that overdose, often accompanied by alcohol and other substances, also contributes to mortality. The method is accurate, sensitive, specific, and precise in detecting cannabis in blood. Blood is preferred for forensic toxicology analysis due to its interpretive value from a pharmacodynamic perspective. Additionally, a wealth of scientific literature references blood drug concentrations [32]. Albeit, the current study has limitations. This method was challenged only by the cannabinoid THC and its metabolite THC—COOH. The lack of reference standards made it impossible to examine other THC isomeric metabolites as potential interferences. To the best of our knowledge, this is the first method to simultaneously analyze THC and THC—COOH in post-mortem blood and urine samples by combining LLE and GC–MS. Previously mentioned methods typically involve extraction techniques that escalate the cost of analysis and necessitate the use of more expensive instruments like LC-MS-MS or GC–MS-MS, which are not practical for routine laboratory use [8,9,13,32,36].

**Table 6:** Summary of authentic samples analysis results that were positive for ∆9-THC, THC—COOH in blood and urine samples.

Case Number	Gender	Age	Cause of Death	Blood	Urine	Other detected substances
				∆^9^-THC ng/mL	THC—COOH ng/mL	
1	M	35	Falling from height	231.04	214.83	–
2	F	29	Drug overdose	144.74	298.74	Amfetamine, Methamfetamine, Methadone,EDDP
3	M	25	Drug overdose	215.65	107.47	Methadone, EDDP, Tramadol, Morphine
4	M	25	Drug overdose	157.26	131.33	Methadone, EDDP, Morphine, Propranolol
5	M	22	Drug overdose	135.8	236.46	Methadone, EDDP, Fluoxetine
6	M	30	Cyanide poisoning	117.73	223.49	–
7	M	25	Car accident	231.25	221.55	Ethanol (90 mg/dL)
8	M	30	Murder (Stabbed)	126.98	55.84	Methadone, EDDP
9	M	21	Drug overdose	193.28	258.10	Methadone, EDDP
10	M	30	Drug overdose	299.03	250.67	Methadone, Alprazolam, Fluoxetine, Nordazepam
11	M	41	Suicidal hanging	99.25	298.80	Morphine, Codeine, Noscapine, Papaverine, Ethanol (10 mg/dl)
12	F	22	Drug overdose	245.66	249.92	Methadone, Diazepam, Morphine, EDDP
13	M	18	Ischemic heart disease	133.39	270.40	Methadone, EDDP
14	M	24	Suicidal hanging	152.34	142.42	Tramadol
15	M	25	Drug overdose	87.9	93.15	Amfetamine, Methamfetamine, Methdone, EDDP, Nordazepam, Diphenhydramine
16	M	20	Drug overdose	269.66	198.47	Methadone, EDDP, Diphenhydramine, Sildenafil
17	M	42	Drug overdose	247.69	185.35	Amfetamine, Methamfetamine, Methadone, EDDP
18	M	22	Suicidal hanging	30.14	233.86	Tramadol, Methadone, EDDP
19	M	25	Myocardial infarction	273.63	96.44	Methadone, EDDP, Ethanol (2 mg/dL)
20	M	31	Drug overdose	290.49	104.80	Methadone, EDDP, Quetiapine, Clomipramine
21	M	35	Natural death	48.73	85.65	–
22	M	19	Drug overdose	24.74	228.15	Methadone, EDDP, Propranolol
23	M	26	Drug overdose	231.25	170.02	Methadone, EDDP, Clonazepam
24	M	23	Firearm suicide	192.41	286.99	Methadone, EDDP, Cyproheptadine
25	M	24	Murder (Stabbed)	115.42	52.91	–
26	M	37	Drug overdose	280.74	265.83	Methadone, EDDP, Pantoprazole, Lidocaine, Mefenamic acid, Ketorolac
27	M	29	Drug overdose	153.34	224.68	Methadone, EDDP, Tramadol, Morphine, Ethanol (7 mg/dL)
28	M	35	Suicidal hanging	25.15	279.23	Chlordiazepoxide, Ethanol (181 mg/dL)
29	F	22	Suicidal hanging	160.15	168.34	–
30	M	21	Myocardial infarction	144.11	155.93	Methadone, EDDP

M: male; F: female. Measured analytes in samples were selected within the validation range.

When collecting postmortem samples in a forensic facility, it's crucial to adhere to specific sensitivity requirements, including sample quantity and storage conditions. Blood samples from deceased individuals need to be carefully stored due to rapid decomposition. This study is the first to examine THC and its metabolite THC—COOH in postmortem blood and urine samples using LLE extraction method and quantitative measurement by gas chromatography-mass spectrometry (GC–MS), highlighting its unique value compared to other studies.

## Conclusions

This study developed a straightforward assay method for evaluating post-mortem blood and urine specimens, involving liquid-liquid extraction (LLE), derivatization with N-methyl-N-(trimethylsilyl)trifluoroacetamide (MSTFA), and gas chromatography-mass spectrometry (GC–MS). The technique was validated for a linear concentration range of 25–300 ng/mL in blood and 50–300 ng/mL in urine. The stability of blood and urine samples was tested at concentrations of 100 and 250 ng/mL at room temperature after 2, 6, and 24 h. Additionally, the stability of the samples was evaluated after three freeze-thaw cycles and by retesting the derivatized extracts. Relative recovery data ranged from 79.74% to 90.80%. The calibration curve's coefficient of determination (R²) was greater than 0.9989 in blood and 0.999 in urine, indicating good linearity. The limit of detection (LOD) was three times the signal-to-noise ratio (S/N), corresponding to 15 ng/mL in blood and 25 ng/mL in urine. The limit of quantification (LOQ), defined as ten times the signal-to-noise ratio (S/N), was 25 ng/mL in blood and 50 ng/mL in urine. Repeatability and reproducibility were assessed for both inter-day (*n* = 3) and intra-day (*n* = 3) measurements. The measured bias ranged as follows: inter-day: −1.56% to 0.13% in urine and −0.07% to 1.34% in blood; intra-day: −2.59% to 0.60% in urine and −2.40% to 6.25% in blood. Precision was estimated as follows: inter-day: 0.70% to 2.08% in urine and 1.51% to 8.43% in blood; intra-day: 0.42% to 8.91% in urine and 0.16% to 11.53% in blood. The developed assay proved to be an efficient method for quantifying THC and THC—COOH concentrations in blood and urine samples and was successfully applied to actual samples as well.

## Limitations

Working in a legal center requires high measurement accuracy. Sample preparation process is a time-consuming step and needs expensive derivatization reagent and high-quality reference standards that make it difficult to achieve.

## Ethics statements

This study was approved by the Legal Medical Center of Tehran Province as part of the investigation into the cause of death. Approval from the Center for Forensic Research and the University of Tehran Ethics Committee was obtained (Approval ID: IR.UT.SPORT.REC.1402.104).

## CRediT authorship contribution statement

**Somayeh Paknahad**: Methodology, Analysis, Validation, Writing – review & editing, **Farzaneh Jokar:** Methodology, Data curation, Supervision, Analysis, Validation, **Mohammad Kazem Koohi:** Methodology, Validation, **Masoud ghadipasha:** Methodology, Validation, **Jalal Hassan:** Methodology, Validation, Analysis, **Maryam Akhgari:** Methodology, Validation, Analysis, Writing—review and editing, **Mehdi Forouzesh:** Methodology, Validation.

## Declaration of competing interest

The authors declare that they have no competing interests or personal relationships that could influence the results of this study.

## Data Availability

Data will be made available on request.
